# Effectiveness of Solution-Focused Group Counseling on the Mental Health of Midwifery Students 

**DOI:** 10.1055/s-0039-1693741

**Published:** 2019-08

**Authors:** Nezhat Javid, Atefeh Ahmadi, Moghadameh Mirzaei, Monavvareh Atghaei

**Affiliations:** 1 Student Research Committee, Kerman University of Medical Sciences, Kerman, Iran

**Keywords:** solution-focused group counseling, mental health, midwifery

## Abstract

**Objective** The present study was conducted with the objective of investigating the effectiveness of solution-focused group counseling (SFGC) on promoting the mental health of midwifery students.

**Methods** The present study is an intervention-based study with a pretest, a post-test, and a control group. The statistical population included all of the midwifery students studying in the midwifery department of the Bam University of Medical Sciences, Kerman, Iran, who filled out the General Health Questionnaire (GHQ) in the screening phase. In the second phase, 40 individuals, having a low level of mental health based on the cutoff score of 23, were selected and randomly divided into 2 groups (intervention and control), each group with 20 participants. The intervention group participated in 5 sessions of 75 minutes for SFGC. Then, the post-test was held in both groups and the data analysis was conducted using the Mann-Whitney and the Kruskal-Wallis test with IBM SPSS Statistics for Windows, Version 21.0 (IBM Corp, Armonk, NY, USA). The significance level was considered as *p* < 0.05.

**Results** The findings showed that the mean of the post-test mental health scores of the intervention group (14.5 ± 50.35) and of the control group (23.6 ± 35.83) showed a statistically significant difference (*p *< 0.0001). Moreover, the comparison between the mean scores of the mental health subscales (physical symptoms, stress, social performance, and depression) showed a statistically significant difference in these groups, and SFGC improved physical symptoms, stress, social performance, and depression in the members of the intervention group.

**Conclusion** Solution-focused group counseling may improve all levels of mental health. This type of counseling is recommended to be used to solve the psychological problems and to improve the mental health of students, as well as of the staff of the health system.

## Introduction

The concept of mental health is, in fact, an aspect of the general concept of health, and refers to all of the methods used to prevent mental diseases, as well as their treatment and rehabilitation.^1^ According to the definition of the World Health Organization (WHO), mental health is a mood of wellbeing that enables individuals to recognize their abilities and to cope with the ordinary stresses of life, as well as to work effectively and efficiently and to help their society.^2^ Also based on the definition of the WHO, mental health refers to the capability of coordinated and harmonic relationship with others, changing and reforming the individual and the social environment and solving personal conflicts and tendencies rationally, fairly, and appropriately.^3^ Individuals with mental health disorders have higher rates of physical diseases, unexpected death due to of coronary artery disease, stroke, diabetes, infections, and respiratory diseases.^4^ Based on the studies conducted in Iran, ∼ 7 million people suffer some kind of mental disorder. Among the common mental disorders in the societies, depression is one of the most common mental disorders that are not limited to a particular time, place or person, and it includes all groups and classes of the society.^5^

Overall, mental disorders are divided into four parts; depression, anxiety, social problems, and related physical symptoms. Studies have shown that out of each four individuals, one person fulfills the diagnostic criteria of at least one stress disorder, and women are more afflicted by them than men.^5^ Individuals with depression and anxiety cannot maintain an effective relationship with others and be compatible with them, and they are prone to a variety of physical diseases.^6^ Individuals who are not absorbed in social frameworks pave the way for growing social deviations and for the development of mental health problems.^7^

As scholars and innovative human resources of any society, university students have a special position; therefore, caring for their mental health along with providing learning and increasing scientific awareness is very important. Entering university is a very critical part in the life of students and is usually followed by many changes in social relationships.^8^

Being in these conditions is often accompanied by pressure and worries, which affects the performance and output of the individual. Moreover, having mental problems leads to difficulties in doing homework, reducing motivation, and increasing anxiety, fear and worries, making students allocate a significant part of their intellectual forces to these problems and, as a result, they will not have the adequate energy and interest in being active for curriculum and educational affairs.^9^ Such a finding was also confirmed in the studies by Rafati et al^8^ and by Ghamari et al.^10^

Due to the educational pressure and to other stressing factors, the students are exposed to different mental disorders, and depression and anxiety can be observed in them in different degrees.^5^ Due to pressuring factors, such as the clinical education environment, encountering the patients, compact courses in internship, the mental and spiritual pressures of the hospital and of the emergency department, as well as facing with the problems of the patients, medical students are more exposed to spiritual and emotional disorders than others.^11^ ^12^ Hashemi Nejad et al^13^ concluded that occupational stresses can induce the development of mental disorders in midwives. Knezevic et al^14^ investigated the working stresses of midwives working in Greek hospitals. In this investigation, they stated that 53% of the midwives considered the reason of their stress work pressure on employees, inadequate income, facing the pain of the patients, and the side effects of the deliveries. Attention to the mental health of midwifery students is of great importance due to their relationship with the health of vulnerable groups (mothers and newborns).^9^ ^15^

According to the WHO, promoting mental health refers to a set of activities that create living conditions and a supportive environment in the field of mental health and that enable individuals to adapt and maintain a healthy lifestyle. These activities increase this possibility for more individuals to experience a better mental health.^16^ Based on several studies, different methods of counseling can improve mental health in people with psychological problems. Solution-focused counseling (SFC) improves mental health by reducing mental pressures, as well as symptoms of anxiety and depression.^17^ ^18^ ^19^ ^20^

Solution-focused counseling is categorized as a postmodern approach to dependent behavioral interventions in the cooperation between therapist and client for receiving treatment.^21^ This approach induces a nonpatient cognition attitude toward the client and helps them to solve their problems.^22^ Unlike the problem-focused attitude, instead of focusing on problems, finding the solutions is emphasized in this approach. Therefore, SFC helps the clients to find the best solution for their issues not solving their problems. Furthermore, it guides them to discover their current potential powers. It leads to increasing hope of disappointed people to the future. Lastly, this method focuses on the solutions not existing problems and their origins in the past.^23^

Midwifery students will be the future expert forces in healthcare that will have an important role in providing care, treatment, and physical and mental support to their patients. Therefore, to have healthy, innovative and empowered midwives, their mental health should be noticed. We have not found any study that investigated the effect of SFC on the promotion of mental health in midwifery students. Regarding the importance of mental health during student life, its effect on the educational achievement and on adaptability of midwifery students, the present research aimed to investigate the effect of SFC on the mental health of midwifery students.

## Methods

The present study is a clinical trial with a pretest and post-test design, as well as intervention and control groups. The statistical population included all 102 midwifery students as the whole of students studying in the midwifery department of the Bam University of Medical Sciences, Kerman, Iran, who filled out the General Health Questionnaire (GHQ) in the screening phase. In the second phase, a total of 40 students, having at least mild mental health problems based on the cutoff score of 23,^24^ ^25^ ^26^ were selected and randomly divided into 2 groups (intervention and control), each group with 20 participants.^27^ The inclusion criteria included studying in the Faculty of Nursing and Midwifery of the Bam University of Medical Sciences, agreement in participating in the study (including signing the informed consent form). The exclusion criteria were patients suffering from chronic, physical and psychological diseases (e.g., asthma, diabetes, major depressive disorder, and bipolar disorder).^28^ ^29^ None of the 40 students were excluded from the study until the end of the research. The intervention group participated in 5 sessions of 75 minutes for solution-focused group counseling (SFGC). No intervention was implemented for the control group, but after the intervention, pamphlets including brief information about the content of the sessions were offered to them. After finishing the sessions, a post-test was held in both groups, and the data were analyzed in IBM SPSS Statistics for Windows, Version 21.0 (IBM Corp., Armonk, NY, USA). To analyze the data, the Mann-Whitney and the Kruskal-Wallis tests were used.

## The Tool for Measuring Mental Health

The GHQ (Appendix A) was developed by Goldberg et al in 1979.^30^ Many studies have confirmed the high validity and reliability of this questionnaire, and it is introduced as a useful tool in diagnosing psychological disorders in the staff of medical sciences.^1^ This questionnaire includes 28 multiple choice questions regarding four fields of physical symptoms: anxiety, social performance disorders, and depression, so that questions 1 to 7 are related to physical symptoms, questions 8 to 14 to anxiety and insomnia, questions 15 to 21 to the domain of social performance disorders, and questions 22 to 28 to depression. The questions have been scored between 0 and 3, based on the 4-degree Likert scale, so that scores ≥ 23 indicate mental disorder, and scores < 23 indicate better mental health.^24^ ^25^ ^26^ ^31^ Nagyova et al^32^ reported the reliability coefficient of this questionnaire and of its subscales (physical symptoms, anxiety, social performance disorders, and depression) through the Cronbach α, as 0.92, 0.83, 0.87, 0.76, and 0.83, respectively. Ebrahimi et al^33^ obtained the reliability of the questionnaire as 0.78. The reliability of this test in a study has been estimated as 0.91 using retesting, and it is reported as 0.88 in another study.^24^ ^26^

**Appendix A:** The 28 items of the scaled version of the General Health Questionnaire (Goldberg et al)^30^

**Have You Recently**
1. Been feeling perfectly well and in good health?
2. Been feeling in need of a good tonic?
3. Been feeling run down and out of sorts?
4. Felt that you are ill?
5. Been getting any pains in your head?
6. Been getting a feeling of tightness or pressure in your head?
7. Been having hot or cold spells?
8. Lost much sleep over worry?
9. Had difficulty in staying asleep once you are off?
10. Felt constantly under strain?
11. Been getting edgy and bad-tempered?
12. Been getting scared or panicky for no good reason?
13. Found everything getting on top of you?
14. Been feeling nervous and strung-up all the time?
15. Been managing to keep yourself busy and occupied?
16. Been taking longer over the things you do?
17. Felt on the whole you were doing things well?
18. Been satisfied with the way you've performed your task?
19. Felt that you are playing a useful part in things?
20. Felt capable of making decisions about things?
21. Been able to enjoy your normal day-to-day activities?
22. Been thinking of yourself as a worthless person?
23. Felt that life is entirely hopeless?
24. Felt that life isn't worth living?
25. Thought of the possibility that you might make away with yourself?
26. Found at times you couldn't do anything because your nerves were too bad?
27. Found yourself wishing you were dead and away from it all?
28. Found that the idea of taking your own life kept coming into your mind?

## Solution-Focused Group Counseling Sessions

The 1^st^ session: initiation of appropriate and trustful relationship, stating the goals of the sessions, trust making, familiarizing and introduction, stating the counseling procedures and regulations of the group, familiarity of the students with the concepts of mental health, SFC, talking about the future, and giving homework for the next session.

The 2^nd^ session: discussing about the homework of the previous session, imagining the problems and proposing solutions for the problem (depression), making participants committed and hopeful to solve problems, identifying and solving the resistances of the participants, using the technique of comparative questions, investigating eliminating solutions, complaints, formulating the trend of problem solving, highlighting and clarifying their capabilities and abilities in problem solving, encouraging the members to investigate the solutions, giving homework for the next session.

The 3^rd^ session**:**checking the homework of the previous session, talking about the future, making students familiar with problem exceptions (anxiety and physical complaints), using exception questions, empowering their capabilities and abilities, summing up the information, and implementing the miracle question.

The 4^th^ session**:** checking the homework of the previous session, helping the participants to identify other ways of thinking, of feeling and of behaving instead of recurrent problematic thinking, feeling and behaving, encouraging the participants to check the solutions, emphasizing on their capabilities and abilities in the field of social performance, using comparative questions, using the master key technique. Giving homework for the next session.

The 5^th^ session**:** a summary of the previous session, checking the homework of the previous session, summing up and conclusion, and determining whether the students have achieved the counseling goals or not. Stating that the solution of problems is in people, and that they can learn and use them; holding post-test.

## Ethical Considerations

The study protocol was reviewed and approved by the Ethics Committees of the Kerman University of Medical Sciences (number: IR.KMU.REC.1396.1180), and the study was registered at the Iranian Registry for Clinical Trials (code: IRCT2017092636443N1).

## Results

All of the students were female, because in Iran only women can be midwives. Table 1 describes the demographic characteristics of the students who participated in the present study.

**Table 1:** Demographic factors

Variable		Intervention group (%) *n*	Control group (%) *n*	*p-value*
Age (years old)	≥ 21	(59.1) 13	(49.9) 9	0.20
	< 21	(38.9) 7	(61.1) 11	
Living status	dormitories	(45) 9	(55) 11	0.52
	house	(55) 11	(45) 9	
Marital status	married	(40) 4	(60) 6	0.46
	single	(53.3) 16	(46.7) 14	
Suffering from chronic physical diseases	yes	0	(100) 1	> 0.99
	no	(51.3) 20	(48.7) 19	
Is interested in the field of the study	yes	(48.5) 16	(51.5) 17	> 0.99
	no	(59.1) 13	(49.9) 9	
Educational status	1^st^ year	(38.9) 7	(61.1) 11	0.34
	2^nd^ year	(45) 9	(55) 11	
	3^rd^ year	(55) 11	(45) 9	
	4^th^ year	(40) 4	(60) 6	

As shown in Table 2, using the Mann-Whitney test and a significance level of 0.05, there is no difference between the control and intervention groups in terms of the pretest score of mental health. Using the Mann-Whitney test, the value of significance for the post-test scores is < 0.05. Therefore, the difference of these groups in the post-test (mental health) is significant, that is, the two groups are different from each other in terms of post-test scores; moreover, it is observed that the post-test score of the intervention group is lower than the score of the control group. Hence, the method used in the intervention group had a significant effect.

**Table 2:** Comparison of the pretest and post-test scores of the of intervention and control groups

Mental health	Control group mean ± SD median	intervention group mean ± SD median	*p-value*
Pretest	29.25 ± 7.65 26	27.20 ± 6.81 25	0.45
Post-test	83.6 ± 35.23 24	14.50 ± 5.35 13.5	< 0.0001

Abbreviation: SD, standard deviation.

Using the Mann-Whitney test, the subscales both in the control and in the intervention group, before the intervention, were compared. Given the significance values shown in Table 3, which are > 0.05, it is concluded that the control and intervention groups are not different in terms of subscales scores (physical symptoms, anxiety, depression, and social performance) before implementing the intervention (pretest). It is concluded that, in the comparison between the two groups in the scores of the subscales (physical symptoms, anxiety, social performance, and depression) after the intervention (post-test), there was a statistically significant difference. It is also observed that the mean value of the subscales in the intervention group was lower than that of the control group, that is, SFGC affected all four subscales and improved the physical symptoms, anxiety, social performance, and depression of the intervention group.

**Table 3:** Comparison of the subscales scores of the intervention and control groups before and after the intervention

Subscale	Before			After		
	Control mean ± SD median	Intervention mean ± SD median	*p-value*	Control mean ± SD median	Intervention mean ± SD median	*p-value*
Physical symptoms	6.70 ± 2.92 6.5	6.70 ± 2.75 5.5	0.46	7.15 ± 3.08 7.5	3.65 ± 2.30 3.5	0.001
Anxiety	8.05 ± 2.08 7.5	8.35 ± 2.47 8	0.65	6.85 ± 2.81 7	3.95 ± 2.37 4	0.001
Social performance	8.65 ± 1.95 8.5	8.45 ± 2.72 9	0.94	7.04 ± 3.20 7.5	5.80 ± 1.70 6	0.049
Depression	5.25 ± 4.83 3	3.70 ± 3.49 3	0.56	1.70 ± 1.34 2	1.15 ± 2.03 0	0.044

Abbreviation: SD, standard deviation.

## Discussion

The importance of mental health and its role in human survival indicates the importance of humans as social creatures. Positive mental health has been a pivotal factor, and it leads to positive recognition, positive behavior, and increasing cognitive abilities.^4^ Unfamiliarity with the university environment at the beginning, being away from the family, lack of interest in the accepted field of study, incompatibility with other people in the living environment, and lack of welfare and economic facilities are some of the conditions that can create or intensify mental problems and lead to a significant decrease in academic achievement.^29^

Given the inevitability of some stressing factors in the midwifery profession and the necessity of preventing the behavioral and mental side effects of stress, assessment of mental health status of midwifery students and training the coping skills in the clinical environment should be the duties of the authorities of healthcare systems.^13^ Given the difficulty of midwifery and the vulnerability of this group, both spiritually and mentally, some measures should be taken to improve the mental health of this vulnerable group of students.

Regarding the aforementioned acceptable psychometric properties of the GHQ,^24^ ^26^ ^33^ this tool was used to investigate the mental health status of the students. The comparison of the results of the tests with the GHQ in the pretest and in the post-test in the present study indicated the effect of the intervention in improving the indicators of mental health of the intervention group in comparison with those of the control group. In other words, group counseling with the solution-focused approach resulted in a reduction of depression, of anxiety, of social problems, and of related physical symptoms.

The results of the Mann-Whitney and of the Kruskal-Wallis tests in the control and intervention groups indicate that there is no statistically significant relationship between demographic variables such as residential area, marital status, interest in the field of study, educational status, and native status of the individuals with the level of their mental health.

The findings of the present study show that SFGC affects positively the mental health of midwifery students, improves their mental health, and that there is a significant difference between the mental health of the students in the intervention group and that of the students in the control group. The total score of all of the participants in the intervention group improved, and only 15%, 10%, and 10% of the scores in the physical symptoms, depression, and social performance domains worsened, respectively.

The findings of the present study are in line with the results of the study by Amiri et al^17^, in which it was concluded that SFC affects the general health of high school student boys with single parents. The main hypothesis of the present study is that SFGC positively affects the mental health of midwifery students. The findings of the present study are in line with the findings of Dastbaz et al,^18^ who stated that SFC is effective in reducing mental pressure. They are also in line with the study by Corcoran,^34^ who concluded, in a comparison between group therapy and ordinary therapy, that group therapy has the most effect in reducing stress and improving attitude and behavior, and with the results of Seagram^35^ about the efficiency of SFGC in improving the attitude, the behavior, and reducing the antisocial behavior and thinking of transgressive adolescents. The results of the present study are in line with the results of other studies, such as the one by Rajabi et al,^36^ about the effect of group counseling with the rational-emotional-behavioral approach on the components of mental health, and with the study by Ghanbari Zarandi et al,^37^ which indicated the effectiveness of group counseling with the approach of meaning therapy on the promotion of the mental health of women who were hurt after an earthquake, as well as with the study by Fakhar et al^38^ about the effectiveness of group counseling with the meaning therapy approach on the level of mental health of elderly women, and with the study by Ahghar,^39^ who indicated the effectiveness of group counseling with the cognitive-behavioral approach on the mental health of student girls .^16^ ^39^

The present study is also in line with the results of the studies by Spilsbury^19^ and by Dahl et al^20^, in which it was concluded that solution-focused short-term therapy reduces the symptoms of depression and anxiety. The results of the present study are also in line with the study by Maljanen et al,^40^ who concluded that short-term solution-focused counseling has been effective in improving depression and anxiety disorders in a 1-year follow-up, and also with the findings of the study by Pomeroy et al,^41^, who indicated the effect of short-term counseling in reducing depression and anxiety.^41^ The results of the present study are also in line with the study by Mireau et al,^42^ who showed that a strengths-based, brief SFC (BSFC) model could increase the number of clients served, while maintaining high-quality services. Based on the results obtained from the present study, it can be said that the individuals who participated in the sessions of SFGC could improve their mental health. It was observed in the present study that SFGC reduces depression in midwifery students, which is in line with the results of Estrada et al,^43^ who investigated the effect of solution-focused counseling on very depressed individuals.

The present study is also in line with the results of Javanmiri et al^44^ and of Lee et al,^45^ who investigated the effects of SFC in reducing depression symptoms. The present study is also in line with the results of the study by Reddy et al,^46^ who concluded that short-term SFC has been effective in improving the depression symptoms of a depressed adolescent girl. It is also in line with the study by Smock et al,^47^ in which SFC has improved the depression symptoms and decreased the distress in depressed addicts.

Investigating the results of the present study, it is concluded that SFGC reduced the physical symptoms of midwifery students, which is in line with the findings of Neilson-Clayton et al,^48^ who could reduce the physical symptoms and the stress of cancer patients through SFC; however, the present study was not in line with the results of Amiri et al^17^ In line with the present study, a systematic qualitative review of controlled outcome studies showed that only 26% of the studies reported a negative benefit from solution-focused behavioral therapy. In addition, the strongest evidence of effectiveness in the present study came in the treatment of depression in adults.^49^ Furthermore, the study by Beyerbach et al indicated that only 18% of the patients stated that their problems were not solved during therapy.^50^

We have also achieved results that indicate that SFGC reduced the anxiety of midwifery students. The results obtained are in line with the results of the study by Wettersten et al^51^, who showed that SFC been able to create positive changes in the rates of stress and of satisfaction of the patients. Our results are also in line with the results of Lambert et al^52^ about the efficiency of SFC in curing the problems of patients with mental disorders, such as mood disorders, anxiety, compatibility disorders, and drug abuse in adults, as well as with the results of the study by Barandeh al^53^ about the effects of this approach in reducing the occupational stress of the staff of the Ghalamchi institution. Our results are also in line with the study of Aslani,^54^ who concluded that SFGC for managing stress decreases stress in pregnant women.

It was observed in the present study that SFGC improved the social performance of midwifery students, which is in line with the results of Sadeghi Shermeh et al,^55^ who studied the effectiveness of solution-focused communication training on the communicational skills of nurses. However, these results are not in line with the studies by Ghanbari Zarandi et al^37^ and by Amiri et al.^17^

The aim of the solution-focused approach is to help the patients to develop solutions, producing a more positive quality of life. This therapy is a future-focused and target-focused approach, concentrating on solutions.^56^ This approach does not look for the pathology of the patients, but assumes them as a person who is stuck with problems.^57^ Instead of an emphasis on the defects and disabilities of individuals, this approach focuses on highlighting the capabilities and successes of individuals and on creating team relations during the treatment.^58^ Based on this treatment, changing and transformation is inevitable, and constructive changes are possible; therefore, in this treatment, the focus in on problems, which are likely to be changed; so, due to this, the treatment is known as hope counselling.^59^ It was shown in many studies that SFGC reduces the mental pressure of students, increases the social compatibility of adolescents, promotes motivation and the progress of the educational performance of individuals, increases the mental health of students, and reduces the depression symptoms, as well as anxiety and stress in different age groups.^17^ ^18^ ^19^ ^20^ ^40^ ^41^ ^46^ ^54^

Maljanen et al^40^ concluded in their study that SFC has been effective in improving the depression and anxiety disorders of hospitalized patients with 1 year of follow-up. Salary Feyzabad^60^ reported that SFGC is effective in reducing mental pressure in high school student girls. The study by Saffarpoor et al^61^ also showed that SFGC has had a significant effect in increasing the social compatibility of orphaned teens. The studies by Alimohammadi^62^ and by Sarvi et al^63^ indicate the efficiency of SFGC in promoting motivation, progress, and educational performance of the target group.^18^ Amiri et al^17^ concluded in their study that SFC increases the mental health of high school student boys with single parents. Spilsbury 19 and Dahl et al^20^ reported that short-term SFC reduces depression and anxiety. Pomeroy et al^41^ reported that short-term SFC is effective in reducing the symptoms of depression and anxiety. Reddy et al^46^ investigated the effect of short-term SFC in improving depression symptoms of a teenage girl with moderate depression symptoms. They showed that the depression symptoms had improved after the treatment sessions.^46^ Aslani et al^54^ concluded in their study that stress management group training with the solution-focused approach reduces the stress in pregnant women.

## Conclusion

The results of the present study show that SFGC affects positively the mental health and its components (physical symptoms, anxiety, social performance and depression) in midwifery students, and improves the mental health and its other components. Based on the results obtained from the present study, it can be said that the individuals who participated in the SFGC sessions could improve their mental health.
